# A comprehensive dataset for the thermal conductivity of ice Ih for application to planetary ice shells

**DOI:** 10.1016/j.dib.2021.107079

**Published:** 2021-04-22

**Authors:** Natalie S. Wolfenbarger, Evan Carnahan, Jacob S. Jordan, Marc A. Hesse

**Affiliations:** a Department of Geological Studies, Jackson School of Geosciences, The University of Texas at Austin, 2305 Speedway, C1160, Austin, TX 78712, USA; b Institute for Geophysics, The University of Texas at Austin, 10100 Burnet Rd, Bldg. 196, Austin, TX 78758, USA; c Oden Institute for Computational Engineering and Sciences, The University of Texas at Austin, 201 E. 24th Street, C0200, Austin, TX 78712, USA; d Department of Earth, Environmental and Planetary Sciences, Rice University, 6100 Main St, Ms-126, Houston, TX 77005, USA

**Keywords:** Thermal conductivity of ice, Temperature dependence of thermal conductivity of ice, Ice thermal conductivity model, Ice thermophysical properties, Ice Ih, thermal conductivity, Ice shell, Uncertainty in ice thermal conductivity

## Abstract

Contemporary models representing the thermal conductivity of ice Ih as a function of temperature are based on data from published experiments that span over a century. Each model is derived using specific datasets with distinct experimental setups, temperature ranges, and uncertainties. Model errors introduced by inaccurate digitization and biased datapoints are challenging to trace due to a lack of transparency of the primary data. This dataset is a collection of published thermal conductivity data for ice Ih, including both tabulated and digitized data, presented in the original units. Specific samples or pressure conditions are noted where applicable. The dataset also includes a survey of published thermal conductivity models, providing the valid temperature range, accuracy and uncertainty (where noted in the original publication), and the primary data sources. Importantly, the dataset includes notes that were contained in the original publication or subsequent publications that provide additional context for the data. This dataset is used to derive a new thermal conductivity model which best represents the thermal conductivity of ice Ih for temperatures greater than 30 K. Statistics are provided to evaluate the fit of each thermal conductivity model in the survey of published models to the comprehensive dataset presented here. This dataset is constructed in support of the work “New insights into temperature-dependent ice properties and their effect on ice shell convection for icy ocean worlds” [1].

## Specifications Table

**Table:** 

Subject	Space and Planetary Science
Specific subject area	Temperature dependence of the thermal conductivity of ice Ih in the ice shells of ocean worlds such as Europa, Enceladus, Titan, and Pluto.
Type of data	Tables
How data were acquired	Data were extracted through transcription of tables and digitization from plots from the following publications: [2,^3^,^4^,^5^,^6^,^7^,^8^,^9^,^10^,11], and [12]. Digitization of datapoints was performed using the open source tool WebPlotDigitizer [13].
Data format	Transcribed Digitized Analyzed
Parameters for data collection	Data included in this collection met the following criteria: (1) Data were referenced in the derivation of published thermal conductivity models for ice Ih (2) Original data source was available for digitization or transcription of datapoints (3) Original data were acquired under atmospheric pressure or could be scaled to atmospheric pressure from higher pressure
Description of data collection	Where data were available as tables, the values were transcribed to the dataset excel file under the tab "K (Tabulated)". Where data were available in the original source as plots, the plots were extracted from the publication using the Snipping Tool available on Windows systems. The image was then uploaded to WebPlotDigitizer and plot axes were calibrated [13]. Datapoints were selected manually and values extracted at a fixed precision of either 2 or 3 digits depending on the units of the data. Data were copied to the dataset excel file under the tab "K (Digitized)".
Data source location	Primary data sources: [2] [3]https://doi.org/10.1080/14786436208209120 [4]https://doi.org/10.1007/978-1-4757-0528-7_32 [5] [6] [7] [8]https://doi.org/10.3189/S0022143000022000 [9] [10]https://doi.org/10.1088/0022-3719/13/4/003 [11]https://doi.org/10.1103/PhysRevB.22.3065 [12]https://doi.org/10.1103/PhysRevB.50.6583
	Sources of thermal conductivity models: [3]https://doi.org/10.1080/14786436208209120 [4]https://doi.org/10.1007/978-1-4757-0528-7_32 [5] [6] [10]https://doi.org/10.1088/0022-3719/13/4/003 [14] [15]https://apps.dtic.mil/dtic/tr/fulltext/u2/a103734.pdf [16]https://doi.org/10.1007/BF01133567 [17]https://pubmed.ncbi.nlm.nih.gov/12148047 [18]https://doi.org/10.1039/B500373C
Data accessibility	Wolfenbarger, Natalie; Carnahan, Evan; Jordan, Jake; Hesse, Marc (2021), “A Comprehensive Dataset for the Thermal Conductivity of Ice Ih for Application to Planetary Ice Shells”, Mendeley Data, V1, doi: 10.17632/ttzbgxs9fw.1https://data.mendeley.com/datasets/ttzbgxs9fw/1
Related research article	Carnahan, E., N. S. Wolfenbarger, Jacob S. Jordan, M. A. Hesse, New insights into temperature-dependent ice properties and their effect on ice shell convection for icy ocean world, Earth Planet. Sci. Lett. (2021). doi: 10.1016/j.epsl.2021.116886

## Value of the Data

• These data are the basis for a number of thermal conductivity models used in a wide array of fields. Papers which derive empirical models from these data often do not explicitly publish their datapoints or present goodness of fit statistics. This reduces the transparency in the models and prevents proper accounting of error or uncertainty, particularly in temperatures ranges where data are sparse.
• Scientists conducting future experiments measuring the thermal conductivity of ice would benefit from comparison to data from previous experiments, which are made more accessible by this dataset. Researchers building numerical and analytical models involving the thermal evolution of ice, including melting and freezing processes, could conduct formal uncertainty quantification of their results by representing the variation in the experimental data of thermal conductivity.
• Comparison of the published datasets and further investigation into differences in experimental setups may support development of experimental procedures that can minimize variation in experimental data by identifying and properly constraining variables that influence thermal conductivity measurements.

## 1 Data Description

File: ice_thermal_conductivity.xlsx

Tabs: K Fits, K (Tabulated), K (Digitized), Supplementary

K Fits: Table which includes a survey of published thermal conductivity models, the range of validity, the published uncertainty/accuracy of the model, the uncertainty/accuracy of the model given all the data collected in this dataset, the source of the model, the source for the original data used to derive the model, and any notes appearing in the literature describing the dataset or the conditions under which it was collected.

K (Tabulated): Table which includes thermal conductivity data transcribed from publications. The source of an individual dataset is provided at the top of the column, followed by the units for temperature and thermal conductivity used in the original publication.

K (Digitized): Table which includes thermal conductivity data digitized from plots presented in publications. Data were digitized using the open source tool WebPlotDigitizer [13]. A fixed precision of two decimal points was used where the data were in units of W/m/K and a fixed precision of three decimal points was used otherwise. The source of an individual dataset is provided at the top of the column, followed by the units for temperature and thermal conductivity used in the original publication. Notes are included where the ice samples were not studied under atmospheric pressure.

Supplementary: Datasets from K (Tabulated) and K (Digitized) used to derive the new comprehensive thermal conductivity model of ice Ih for temperatures greater than 30 K in support of the work “New insights into temperature-dependent ice properties and their effect on ice shell convection for icy ocean worlds” [1].

## 2 Experimental Design, Materials and Methods

Data were collected from published studies of the thermal conductivity of ice Ih as a function of temperature. Because the intention was to use these data to obtain a thermal conductivity model for the ice shells of ocean worlds, datasets that spanned different portions of the relevant temperature range (>30 K) were included [19]. A fixed precision (typically 2 or 3 digits) was specified when exporting the data from WebPlotDigitizer [13] to ensure that the extracted value was insensitive to minor misalignments in datapoint selection (i.e., a precision was adopted such that the value of the datapoint was stable over the range of pixels where the datapoint was defined).

The thermal resistivity and conductivity datasets of [10] and [12] are published at isochoric conditions. The authors scale their data to isochoric conditions for comparison to theoretical phonon scattering models which do not account for changes in volume that occur with temperature. Most of the thermal conductivity data included in this dataset are obtained at isobaric conditions at 1 atm. To obtain a fit of thermal conductivity that incorporates the datasets of [10] and [12], corrections must be applied to the datasets to scale them to isobaric conditions at atmospheric pressure. Both [10] and [12] published the constants which they used to scale their datasets to isochoric conditions at a reference temperature. The data of [12] obtained at 0.08 GPa and 0.16 GPa were scaled to atmospheric pressure using $\frac{\partial \ln r}{\partial P}=0.28\;\text{GP}a^{-1}$, where r is the thermal resistivity (the reciprocal of thermal conductivity) and P is the pressure. The data of [10] were corrected from isochoric to isobaric conditions by accounting for the change in density expected with temperature using $\frac{\partial \ln r}{\partial \ln V}=-2.6$ and the volumetric thermal expansion coefficient for ice as a function of temperature obtained by [20] presented in [14]. The MATLAB script used to perform these corrections and derive the thermal conductivity model in support of the work “New insights into temperature-dependent ice properties and their effect on ice shell convection for icy ocean worlds” is supplied alongside the thermal conductivity dataset. Both publications noted that the maximum correction was 4% from isobaric to isochoric which was in-family with the calculated correction applied here, although the correction applied to the data of [12] for this dataset was closer to 4.5%.

## Ethics Statement

Not applicable.

## CRediT Author Statement

**Natalie S. Wolfenbarger:** Conceptualization, Methodology, Software, Formal analysis, Investigation, Data curtion, Writing – original draft, Writing – review & editing, Visualization; **Evan Carnahan:** Conceptualization, Formal analysis, Investigation, Writing – review & editing; **Jacob S. Jordan:** Writing – review & editing; **Marc A. Hesse:** Conceptualization; Supervision, Writing – review & editing.

## Declaration of Competing Interest

The authors declare that they have no known competing financial interests or personal relationships which have or could be perceived to have influenced the work reported in this article.
